# Cardiac amyloidosis is prevalent in older patients with aortic stenosis and carries worse prognosis

**DOI:** 10.1186/s12968-017-0415-x

**Published:** 2017-12-07

**Authors:** João L. Cavalcante, Shasank Rijal, Islam Abdelkarim, Andrew D. Althouse, Michael S. Sharbaugh, Yaron Fridman, Prem Soman, Daniel E. Forman, John T. Schindler, Thomas G. Gleason, Joon S. Lee, Erik B. Schelbert

**Affiliations:** 1 Department of Medicine, University of Pittsburgh School of Medicine, Pittsburgh, Pennsylvania, 200 Lothrop Street, Scaife Hall S-558, Pittsburgh, PA 15213 USA; 2 UPMC Cardiovascular Magnetic Resonance Center, Heart and Vascular Institute, Pittsburgh, Pennsylvania, USA

**Keywords:** Aortic Stenosis, Cardiac Amyloidosis, Outcomes, Cardiovascular magnetic resonance

## Abstract

**Background:**

Non-invasive cardiac imaging allows detection of cardiac amyloidosis (CA) in patients with aortic stenosis (AS). Our objective was to estimate the prevalence of clinically suspected CA in patients with moderate and severe AS referred for cardiovascular magnetic resonance (CMR) in age and gender categories, and assess associations between AS-CA and all-cause mortality.

**Methods:**

We retrospectively identified consecutive AS patients defined by echocardiography referred for further CMR assessment of valvular, myocardial, and aortic disease. CMR identified CA based on typical late-gadolinium enhancement (LGE) patterns, and ancillary clinical evaluation identified suspected CA. Survival analysis with the Log rank test and Cox regression compared associations between CA and mortality.

**Results:**

There were 113 patients (median age 74 years, Q1-Q3: 62–82 years), 96 (85%) with severe AS. Suspected CA was present in 9 patients (8%) all > 80 years. Among those over the median age of 74 years, the prevalence of CA was 9/57 (16%), and excluding women, the prevalence was 8/25 (32%). Low-flow, low-gradient physiology was very common in CA (7/9 patients or 78%). Over a median follow-up of 18 months, 40 deaths (35%) occurred. Mortality in AS + CA patients was higher than AS alone (56% vs. 20% at 1-year, log rank 15.0, *P* < 0.0001). Adjusting for aortic valve replacement modeled as a time-dependent covariate, Society of Thoracic Surgery predicted risk of mortality, left ventricular ejection fraction, CA remained associated with all-cause mortality (HR = 2.92, 95% CI = 1.09–7.86, *P* = 0.03).

**Conclusions:**

Suspected CA appears prevalent among older male patients with AS, especially with low flow, low gradient AS, and associates with all-cause mortality. The importance of screening for CA in older AS patients and optimal treatment strategies in those with CA warrant further investigation, especially in the era of transcatheter aortic valve implantation.

## Background

Cardiac amyloidosis (CA), especially from wild-type transthyretin-related CA (wtATTR), may be prevalent in older male patients with aortic stenosis (AS) and promote increased risk of mortality. The prevalence of CA [1] and aortic stenosis both increase with age [2–7]. Transcatheter aortic valve replacement (TAVR) expands the pool of patients eligible for treatment of AS [8], further emphasizing the need to understand the prevalence of CA in AS and its prognostic associations. For example, CA may prevent patients from obtaining survival benefit from aortic valve replacement (AVR). Clinically, discerning moderate from severe AS and defining optimal treatment strategies for these groups can be challenging, especially when valve gradients are not severely increased or when severity measures are discrepant. Furthermore, heart failure symptoms may trigger AVR in AS, but may be actually be attributable to clinically unsuspected CA. The recognition of concomitant CA in patients with AS may improve patient stratification and thereby better inform shared decision making and management choices.

Once thought to be a rare type of infiltrative cardiomyopathy due to interstitial expansion from insoluble misfolded proteins, CA has been increasingly diagnosed due to the advances in non-invasive cardiac imaging [9, 10]. Cardiovascular magnetic resonance imaging (CMR) employing late-gadolinium enhancement imaging (LGE) detects interstitial expansion associated with CA, [11, 12] where the presence, pattern and extent of LGE appear prognostically important [13–15].

To date, no studies have reported the prevalence of CA detected by LGE in the denominator of older patients with moderate and severe AS, and importantly its association with outcomes while accounting for other confounders. To address these issues, we retrospectively identified consecutive AS patients defined by echocardiography referred for further CMR assessment of valvular, myocardial, and aortic disease. We examined the prevalence of CA and its associations with age, gender and all-cause mortality.

## Methods

This retrospective single center cohort study was approved by the University of Pittsburgh Institutional Review Board Committee with a waiver of individual consent. We studied consecutive patients with moderate and severe AS by transthoracic echocardiogram (TTE) who also received clinical CMR between 2012 through 2015. The indications for CMR performance were: a) bicuspid aortic valve stenosis with or without aortopathy (*n* = 30), b) AS + left ventricular (LV) dysfunction to evaluate myocardial fibrosis/viability pre-intervention (*n* = 49), c) AS and red flags observed on TTE concerning for CA such as increased biventricular wall thickness, poor longitudinal annular motion and restrictive filling pattern (*n* = 21), d) evaluation of AS severity and TAVR planning (*n* = 7), e) evaluation of myocardial ischemia (*n* = 6). Electronic medical record review identified baseline characteristics, New York Heart Association functional class, and vital status. Hypertension, diabetes, dyslipidemia and prior myocardial infarction were verified according to the information recorded in the electronic medical records.

### Transthoracic echocardiogram

A comprehensive TTE was performed according to the guidelines [16] in accredited Echocardiography Lab by the Intersocietal Commission for the Accreditation of Echocardiography. The pulsed-wave Doppler transmitral inflow velocities and tissue Doppler-derived mitral annular velocities were obtained from apical 4-chamber views for assessment of diastolic function in accordance with the societal guidelines (i.e., pulsed-wave Doppler-derived transmitral inflow velocities of the early phase (E) and late phase (A) of diastole and pulse-wave tissue Doppler-derived mitral annular velocity imaging for both septal and lateral walls) [17]. The E/e’ ratio of early diastolic filling/tissue Doppler velocity annulus was used as the surrogate of LV filling pressures. Longitudinal LV systolic function was also obtained by measurement of the peak systolic velocity from both medial and lateral annuli. Spectral Doppler of the LV outflow tract (LVOT, pulse wave) and aortic valve (AV, continuous wave) were measured using the best baseline view determined for Doppler assessment. For each Doppler measurement 3 cycles were averaged and post–premature ventricular contraction beats were discarded (5 cycles were averaged for patients with atrial fibrillation). Aortic valve area (AVA) was calculated by using the continuity equation formula: AVA = (LVOT area x LVOT VTI)/AV VTI [18] (LVOT = left ventricular outflow tract; VTI = velocity time integral). Stroke volume was calculated by Doppler using the VTI of the LVOT and its diameter in midsystole in the parasternal long-axis view. Stroke volume index was calculated by indexing stroke volume to body surface area. Low-flow, low-gradient AS was defined by stroke volume index <35 ml/m^2^ and aortic valve mean gradient was <40 mmHg. Severe AS was defined as an indexed AVA ≤ 0.6 cm^2^/m^2^ [19].

### Cardiovascular magnetic resonance scans

CMR was performed with a 1.5 T CMR system (Magnetom Espree Siemens Healthineers, Erlangen, Germany) using a 32-channel phased array cardiovascular coil in a CMR laboratory accredited by the Intersocietal Commission for the Accreditation of Magnetic Resonance Laboratories employing dedicated CMR technologists. Balanced steady-state free precession cine images were acquired (slice thickness 6 mm, 4 mm gap) during 5 to 10-s breath holds in the usual short and long axis orientations. CMR assessment of LV volumes, LV ejection fraction (LVEF), LV mass was performed by manual tracing of the endocardial borders at end-diastole and end-systole in each of the short-axis slices as per standard clinical evaluation.

### Late gadolinium enhancement and identification of CA

To identify CA as part of the CMR, LGE imaging was performed 10–15 min after a 0.2 mmol/kg IV gadoteridol bolus (Prohance, Bracco Diagnostics, Princeton, New Jersey, USA) using phase-sensitive inversion recovery (PSIR) pulse sequence which recently has been shown to be an accurate method to identify CA and prognostically important [14]. We identified CA by CMR when LGE imaging demonstrated characteristic pattern of marked myocardial enhancement (either subendocardial or transmural) with associated morphologic findings such as increased LV wall thickness and abnormal myocardial and blood pool kinetics [11, 14]. Applying similar classification used by Fontana et al., a patient with basal transmural LGE but mid/apical subendocardial LGE would be classified as having transmural LGE [14].

Given the retrospective nature of this report, patient’s advanced age, frailty and multiple comorbidities, AL (light-chain) amyloidosis was not systematically excluded via invasive cardiac biopsy or 99^m^–Tc-pyrophosphate nuclear scan (introduced at our center in late 2015), which has recently shown to be an excellent non-invasive method to confirm wtATTR CA [20]. Nonetheless, primary CA was attempted to be excluded based on negative bone marrow and fat-pad biopsies (1 patient); positive 99^m^–Tc-pyrophosphate nuclear scan, Perugini grade 3, which is more specific for wtATTR (1 patient) and negative serum/urine immunoelectrophoresis and immunofixation for paraproteins (4 patients). All imaging studies were analyzed and interpreted by experienced CMR readers, blinded to the clinical outcomes. CMR results were reported to the clinicians caring for the patients.

### Native T1 mapping and extracellular volume fraction

A subset of patients with AS and with AS + CA had native T1 mapping and Extracellular Volume Fraction (ECV) available for measurement. As previously reported by our group [21], T1 mapping was obtained using breath-held modified Look-Locker inversion recovery (MOLLI) sequence after generation of in-line parametric mapping of the basal and mid LV short-axis slices, averaging measures from the middle third of the myocardium, to avoid partial volume effects. Regions of interest excluded myocardium with myocardial infarction and carefully avoided myocardium near infarcted myocardium. We did not exclude foci of nonischemic scar on LGE images (ie, atypical of myocardial infarction) from ECV measures acquired in noninfarcted myocardium. Quartiles of native T1 mapping and ECV values were tested for their association with all-cause mortality and stratified according to the treatment received (AVR vs. medical therapy).

### Pre-aortic valve intervention risk stratification

Surgical Thoracic Society Predicted Risk of Mortality (STS-PROM) was calculated for each patient according to the planned treatment (i.e.: AVR +/− coronary artery bypass grafting) using Society of Thoracic Surgery online calculator (version 2.73) which is a well-validated composite score comprised of over 40 clinical parameters and risk-factors.

### Statistical analysis

Baseline demographic data and clinical variables were summarized with continuous variables expressed as mean ± SD and categorical data presented as frequency (percentage). Differences between the groups were compared with the Student’s t test for continuous variables and the chi-square test for categorical variables. AVR performed either via surgical or TAVR method was considered as a time-dependent covariate. The primary end-point was all-cause mortality after CMR study. Univariable models tested the association of clinical risk factors and imaging findings to all-cause mortality. Multivariable Cox regression models were used to assess the relationship between wtATTR and all-cause mortality. Statistical analysis was performed using SAS software (version 9.4, SAS Institute, Cary, North Carolina, USA).

## Results

### Prevalence of CA

**Fig. 1:**
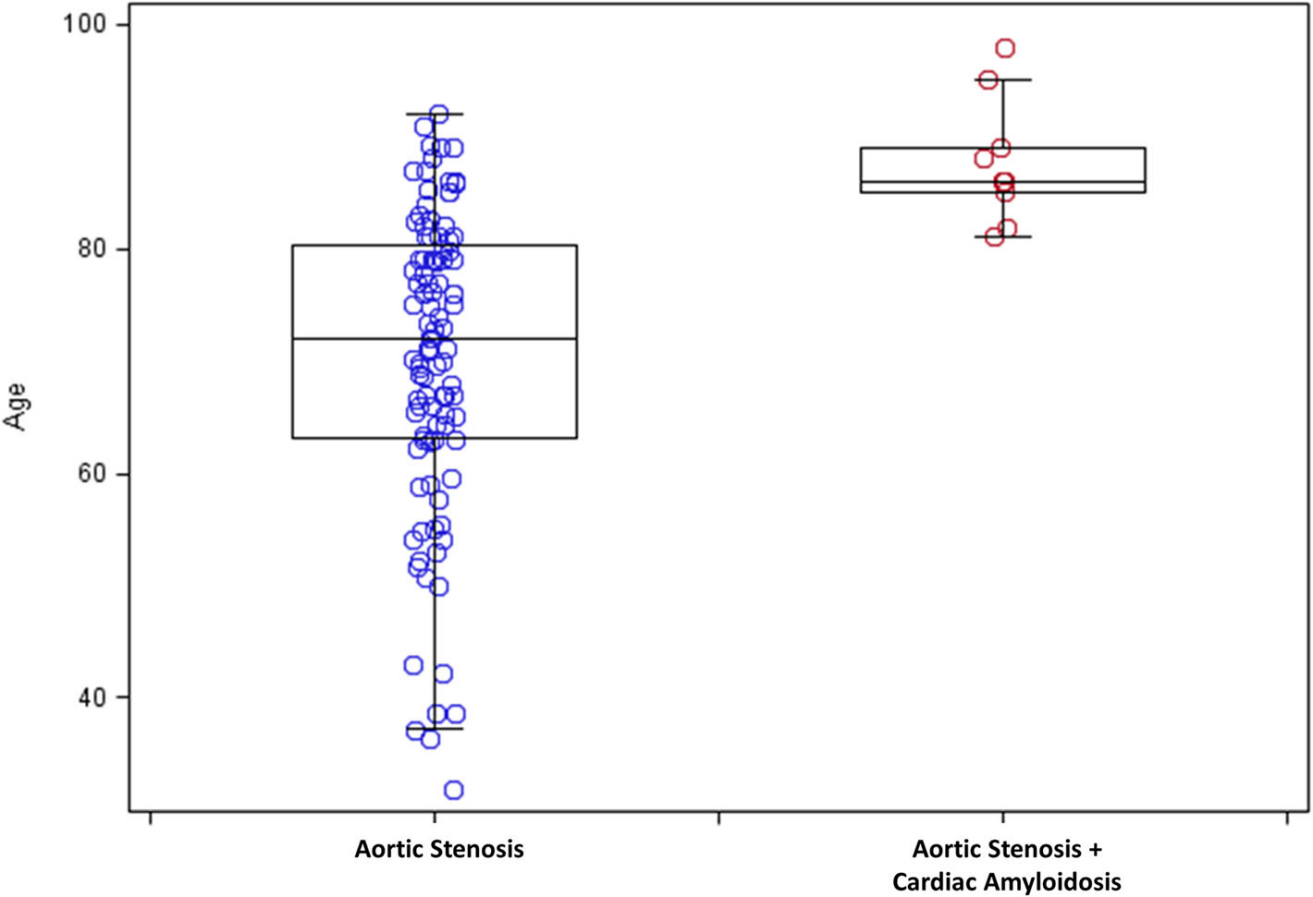
Box and Whisker Plot of Patient Age distribution between aortic stenosis (AS) vs AS + cardiac amyloidosis (CA)

A total of 113 consecutive patients with moderate and severe AS (59 males, median age 74 years, interquartile range: 62–82 years) were studied (96/113, 85% with severe AS). AS combined with CA was present in 9 patients (8%, all >80 years; 8/9 males). The median time interval between the clinical CMR study and TTE study was 6 days (interquartile range of 0–15 days). Baseline clinical and imaging characteristics are summarized in Table 1. The average age for patients with CA was higher than those with isolated AS (88 ± 6 vs. 70 ± 14, *P* < 0.0001, Fig. 1). Among those over the median age of 74 years, the prevalence of CA was 9/57 (16%), and after excluding women, the prevalence was 8/25 (32%). In our cohort, 1 out of 4 male octogenarians presenting with symptomatic AS were found to have concomitant CA detected by CMR.

**Table 1:** Baseline clinical and imaging characteristics

		Aortic stenosis (*N* = 104)	AS + CA (*N* = 9)	*P* Value
Clinical	Age (years)	70 ± 14	88 ± 6	< 0.001
	Male Gender	58 (56%)	8 (89%)	0.057
	Hypertension	76 (73.1%)	7 (77.8%)	0.866
	Diabetes	36 (34.6%)	3 (33.3%)	0.890
	Creatinine (mg/dl)	1.26 ± 0.94	1.54 ± 0.45	0.380
	Prior Revascularization (PCI or CABG)	33 (31.7%)	3 (33.3%)	0.968
	NYHA Class ≥ III at baseline	57 (55%)	7 (78%)	0.182
	Atrial Fibrillation/Flutter	21 (20.2%)	6 (67%)	0.006
	STS Predicted Risk of Mortality (STS PROM) (%)	3.8 ± 3.7	6.9 ± 4.2	0.024
	Any AVR (Surgical or Transcatheter)	55 (53%)	4 (44.4%)	0.627
Echocardiographic	Interventricular Septal Thickness (cm)	1.3 ± 0.3	1.8 ± 0.5	< 0.001
	Relative Wall Thickness (PWT/LVEDD)	0.5 ± 0.3	0.7 ± 0.3	0.147
	Left Atrial Volume Index (ml/m2)	40 ± 15	51 ± 13	0.037
	Septal s’ (cm/s)	4.8 ± 1.7	2.9 ± 1.0	0.008
	Septal e’ (cm/s)	4.9 ± 2.0	3.5 ± 1.2	0.084
	E/e’ ratio (Lateral)	18 ± 11	19 ± 4	0.942
	E/e’ ratio (Septal)	25 ± 18	33 ± 10	0.281
	LV Stroke Volume Index (ml/m^2^)	37 ± 12	25 ± 7	0.003
	Indexed Aortic Valve Area (cm^2^/m^2^)	0.5 ± 0.2	0.4 ± 0.2	0.047
	Severe AS (indexed AVA ≤ 0.6 cm^2^/m^2^)	80 (77%)	8 (89%)	0.43
	AV Mean Gradient (mmHg)	31 ± 15	30 ± 14	0.924
	Low-Flow, Low-Gradient Physiology^a^ (%)	47 (45%)	7 (78%)	0.060
	Pulmonary Artery Systolic Pressure (mmHg)	41 ± 13	45 ± 17	0.435
CMR	LV End-Diastolic Volume Index (ml/m^2^)	92 ± 33	82 ± 19	0.113
	LV End-Systolic Volume Index (ml/m^2^)	48 ± 33	49 ± 22	0.131
	LV Stroke Volume Index (ml/m^2^)	44 ± 13	33 ± 10	0.024
	LV Ejection Fraction (%)	52 ± 18	43 ± 17	0.176
	LV Mass Index (g/m^2^)	73 ± 21	105 ± 21	< 0.0001
	LV Mass/Volume Ratio (LV Mass/LVEDV)	0.8 ± 0.2	1.3 ± 0.3	0.02
	Native T1 mapping (msec)^b^	1035 ± 60	1125 ± 49	0.002
	Extracellular Volume Fraction (ECV) (%)^c^	27.9 ± 4.1	41.2 ± 16.7	< 0.001

Continuous variables are presented as mean ± standard deviation

(^a^) Defined as LV stroke volume index <35 ml/m2 and mean AV gradient <40 mmHg. (^b^) Native T1 available in 74/104 AS patients and in 7/9 AS + CA patients). (^c^) ECV values available in 66/104 of AS patients and 5/9 patients with AS + CA)

*AS* aortic stenosis, *AVR* aortic valve replacement, *CA* cardiac amyloidosis, *CABG* coronary artery bypass grafting, *CMR* cardiovascular magnetic resonance, *ECV* extracellular volume fraction, *LV* left ventricular, *LVEDV* left ventricular end-diastolic volume, *NYHA* New Yo0rk Heart Association, *PCI* percutaneous coronary intervention

### Comorbidities and imaging findings of CA patients

Patients with CA also had on baseline TTE larger left atrial volumes, smaller indexed AVA, and poor longitudinal function by tissue Doppler. The prevalence of severe AS was not statistically different between groups (77% vs 89%, *P* = 0.43, Table 1). Consistently, the CMR showed pronounced concentric LV remodeling in CA with higher LV mass index, higher mass/volume ratio and lower stroke volume index. The average LVEF trended lower in CA patients (43 ± 17% vs. 52 ± 18%, *P* = 0.18).

**Fig. 2:**
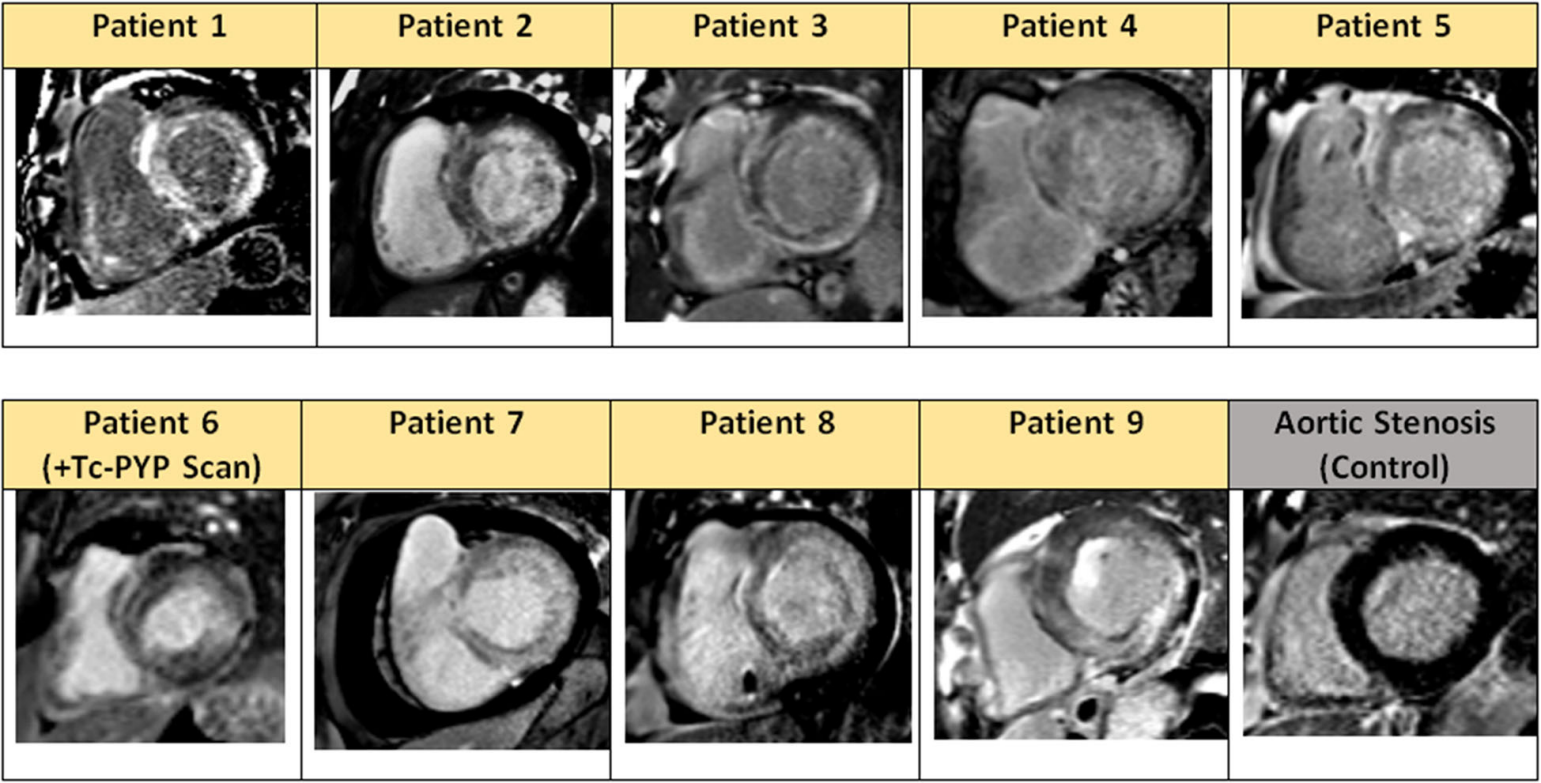
Late gadolinium enhancement (LGE) detection of CA using phase-sensitive reconstruction inversion recovery. Representative basal short-axis image of the 9 CA cases and an AS patient are depicted

Native T1 and ECV values were available in 72% and 63% of the cohort, respectively, and significantly higher in AS + CA patients, when compared to patients with only AS. (Table 1). At our center, normal values for native T1 mapping and ECV for healthy controls are 1016 ± 28 msec and 23.7 ± 2%, respectively [22]. STS-PROM was also higher in the AS and CA patients (6.9 ± 4.2% vs. 3.8 ± 3.7%, *P* = 0.02), consistent with higher surgical risk and greater burden of comorbidities. There was a high burden of atrial fibrillation in CA patients compared to isolated AS (67% vs 20%, *P* = 0.006). Of note, none of the CA patients met electrocardiographic criteria for low voltage. On the other hand, low-flow, low-gradient AS physiology was very common in CA (7/9 patients or 78% vs. 47/104 or 45% with AS, *P* = 0.06) (Table 2). On LGE imaging, all 9 patients with CA demonstrated typical transmural myocardial involvement as shown in Fig. 2.

**Table 2:** Clinical, Imaging Characteristics, Treatment and Outcomes of Patients with AS + Cardiac Amyloidosis

	Patient 1	Patient 2	Patient 3	Patient 4	Patient 5	Patient 6	Patient 7	Patient 8	Patient 9
ECG Rhythm	AFib	AFib	AFib	AFib	NSR	NSR	S. Tach	AFib	AFib
Low Voltage Criteria	No	No	No	No	No	No	No	No	No
Echocardiographic findings									
IVS/IL Thickness (cm)	2.0/1.9	1.7/1.5	1.7/1.4	1.9/1.5	1.8/1.5	1.9/1.5	1.6/1.6	2.1/1.9	1.6/1.6
Aortic Valve Area (cm^2^)	0.3	0.7	0.4	0.9	0.6	0.8	0.6	1.1	0.5
Mean AV Gradient (mmHg)	18	33	33	13	30	47	37	12	50
Stroke Volume Index (ml/m^2^)	16	32	27	22	28	35	14	21	37
CMR Findings									
LV Ejection Fraction (%)	23	45	35	53	35	67	18	49	51
LV End-Diastolic Vol Index (ml/m^2^)	104	64	93	60	94	47	91	88	101
LV Mass Index (g/m^2^)	101	103	97	113	108	73	84	139	128
Native T1 (msec)	1127	N/A	1115	1141	1097	1091	1081	1225	N/A
ECV (%)	70	N/A	40	33	28	35	N/A	46	N/A
Treatment and outcomes									
Aortic Valve Replacement	No	No	TAVR	No	TAVR	No	No	TAVR	TAVR
Status	Dead	Dead	Dead	Alive	Alive	Alive	Dead	Alive	Dead
Survival after CMR (months)	1	2	3	6	8	8	3	5	2

*IVS* interventricular septum thickness, *IL* inferolateral wall, *ECV* extracellular volume fraction, *N/A* Not available. *ECG* electrocardiogram, *TAVR* transcather aortic valve replacement

Table 2 provides a detailed characterization of these 9 patients identified with AS + CA.

### Outcomes

**Fig. 3:**
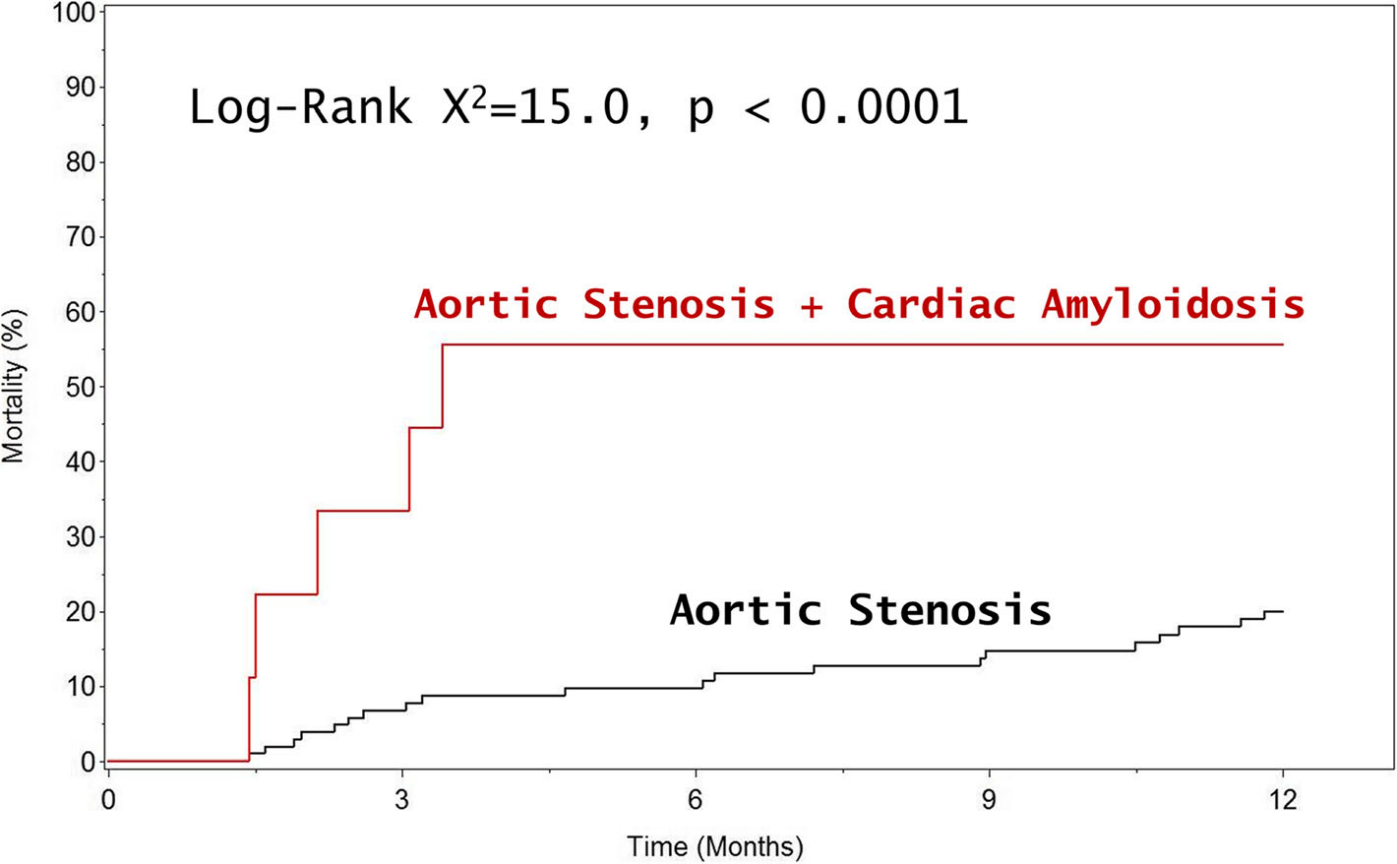
Kaplan-Meier Curves Comparing All-Cause Mortality in AS vs. AS + CA Patients

Over the follow-up period (median 18 months, interquartile range [IQR]: 11–30 months), 59 patients received an AVR (42 surgical AVR and 17 a TAVRs), and 40 patients (35%) died. Patients with CA had significantly higher 1-year all-cause mortality than patients with isolated AS (56% vs. 20%, *P* < 0.001, Fig. 3). Among patients with isolated AS, 55/104 (53%) received AVR and among patients with CA, 4/9 (44%) received AVR, all of them transcatheter AVR.

Univariate analysis of all-cause mortality showed several associations between mortality and patient characteristics (Table 3). Limited events constrained the number of variables that could be included in the multivariable Cox regression model (10 events per predictor variable). Hence, multiple models were created to determine whether CA was a risk factor associated with mortality when adjusting for potential confounders. After adjustment for AVR, STS PROM, LVEF, NYHA Class ≥ III at presentation, the presence of CA remained a predictor of all-cause mortality with similar hazard ratio (Table 4, Models 1 through 3). Since CA was only present in relatively older AS patients, a subgroup analysis was performed in the subgroup aged greater than the total population’s median age of 74 years. Within this older subgroup, the presence of CA, remained a predictor of all-cause mortality with nearly 3-fold increased risk (HR = 2.87, 95% CI 1.02–8.05, *P* = 0.04).

**Table 3:** Univariate Clinical and Imaging Predictors of All-Cause Mortality

	Variable	X^2^	HR (95% CI)	P Value
Clinical	Age (per 1 year)	10.6	1.05 (1.02, 1.08)	0.001
	Male Gender	7.8	2.89 (1.37, 6.10)	0.005
	NYHA Class ≥ III	9.1	3.18 (1.50, 6.73)	0.003
	Hypertension	0.35	1.26 (0.58, 2.74)	0.55
	Diabetes	1.72	1.52 (0.81, 2.83)	0.19
	Prior Coronary Revascularization	8.1	2.48 (1.33, 4.65)	0.004
	Atrial Fibrillation/Flutter	4.56	2.07 (1.06, 4.03)	0.03
	Any AVR (vs. Medical Therapy)	12.0	0.30 (0.15, 0.59)	0.001
	STS PROM (per 1% increase)	21.5	1.15 (1.09, 1.23)	< 0.001
Echocardiographic	Interventricular Septal Thickness (per 1 cm)	4.1	3.14 (1.03, 9.56)	0.04
	Left Atrial Volume Index (per 1 ml/m^2^)	20.7	1.04 (1.02, 1.06)	< 0.001
	LV Stroke Volume Index (per 1 ml/m^2^)	10.8	0.95 (0.92, 0.98)	0.001
	Septal s’ (per 1 cm/s)	2.65	0.77 (0.56, 1.06)	0.10
	E/e’ ratio (lateral)	3.1	1.03 (0.99, 1.06)	0.08
	E/e’ ratio (septal)	6.6	1.02 (1.006, 1.04)	0.01
	AV Peak Velocity (per 1 m/s)	2.76	0.71 (0.48, 1.06)	0.097
	AV Mean Gradient (per 1 mmHg)	2.2	0.98 (0.96, 1.00)	0.13
	Aortic Valve Index (per 1 cm^2^/m^2^)	3.8	0.12 (0.01, 1.00)	0.05
	Dimensionless Index	1.2	0.14 (0.005, 4.32)	0.26
	Pulmonary Artery Systolic Pressure (per 1 mmHg)	10.0	1.03 (1.01, 1.06)	0.002
CMR	Presence of Cardiac Amyloidosis on CMR	8.1	4.10 (1.55, 10.84)	0.004
	LV Ejection Fraction (per 1%)	17.6	0.96 (0.95, 0.98)	< 0.001
	LV End-Diastolic Volume Index (per 1 ml/m^2^)	6.8	1.01 (1.003, 1.02)	0.009
	LV End-Systolic Volume Index (per 1 ml/m^2^)	10.8	1.01 (1.006, 1.02)	0.001
	LV Stroke Volume Index (per 1 ml/m^2^)	4.4	0.97 (0.94, 0.99)	0.037
	LV Mass Index (per 1 g/m^2^)	10.2	1.02 (1.008, 1.04)	0.001
	LV Mass/Volume Ratio	0.006	1.05 (0.30, 3.70)	0.937

**Table 4:** Multivariate cox-proportional hazards models

	Model 1 (X^2^ = 43.8, *P* < 0.0001)			Model 2 (X^2^ = 53.2, *P* < 0.0001)			Model 3 (X^2^ = 51.7, *P* < 0.0001)		
	HR	95% CI	*P* value	HR	95% CI	*P* value	HR	95% CI	*P* value
Cardiac amyloid (vs. No Cardiac amyloid)	2.80	(1.05, 7.5)	0.04	2.84	(1.07, 7.56)	0.037	2.95	(1.08–8.03)	0.035
Any AVR (vs. Medical therapy)	0.29	(0.15, 0.58)	< 0.001	0.33	(0.16, 0.65)	0.001	0.22	(0.11, 0.46)	< 0.001
STS PROM (per 1%)	1.16	(1.09, 1.24)	< 0.001	1.17	(1.09, 1.26)	< 0.001	1.15	(1.07, 1.23)	< 0.001
CMR LVEF (per 1%)	–	–	–	0.97	(0.95, 0.98)	< 0.001	–	–	–
NYHA Class ≥ III at presentation	–	–	–	–	–	–	3.63	(1.68, 7.82)	0.001

**Fig. 4:**
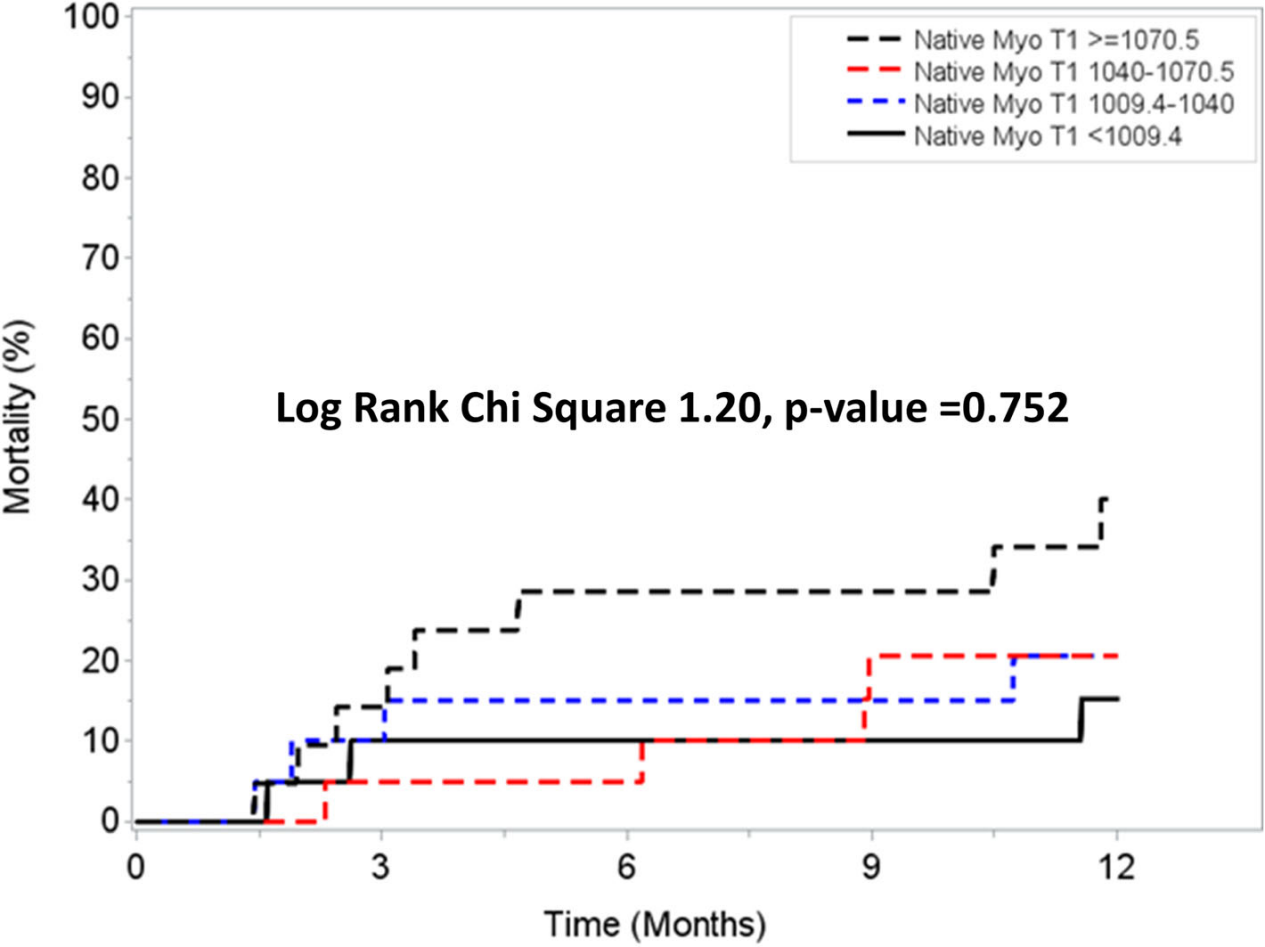
One year survival according to Native T1 quartiles

**Fig. 5:**
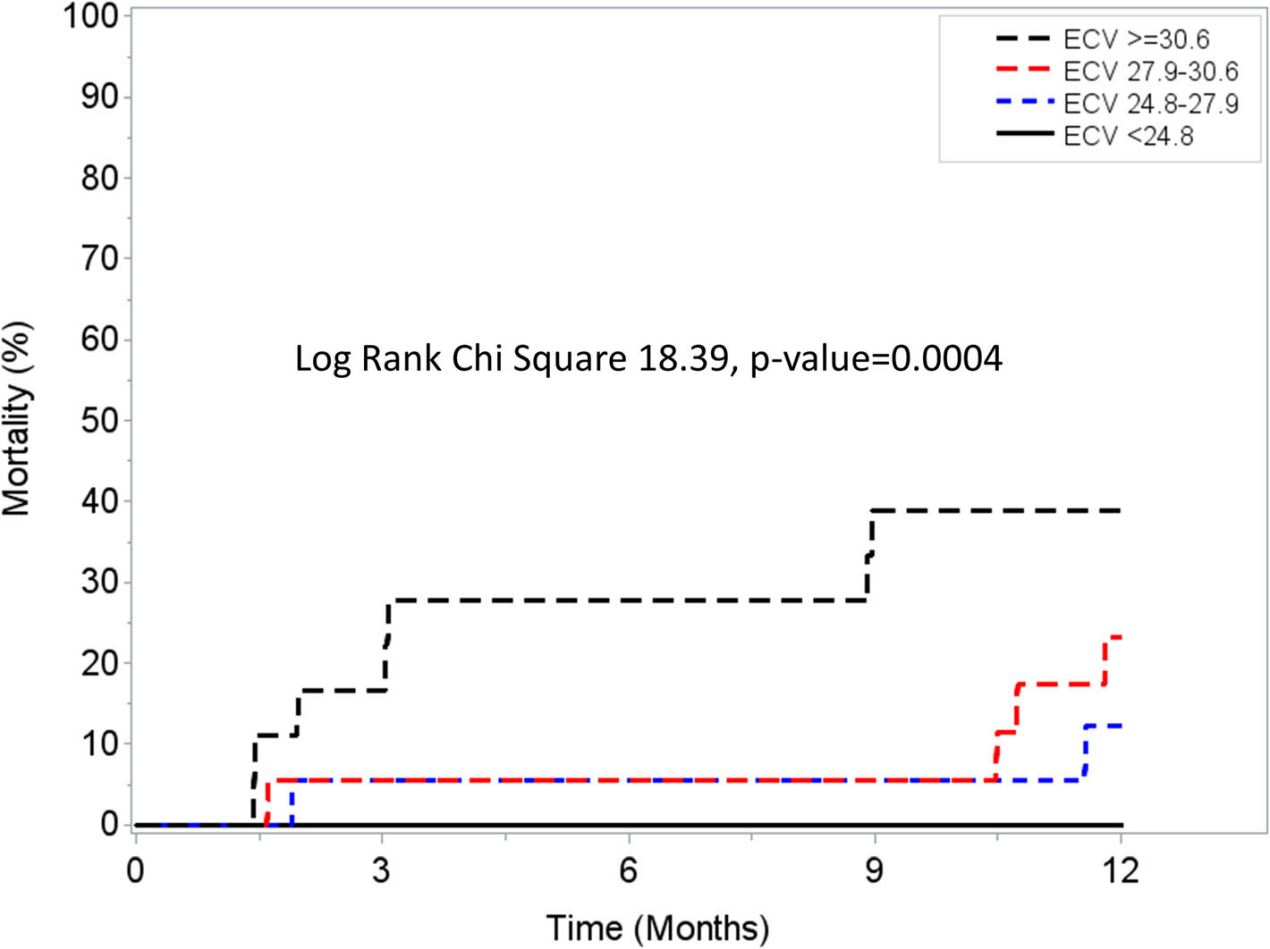
One year survival according to extracellular volume fraction (ECV) quartiles

**Fig. 6:**
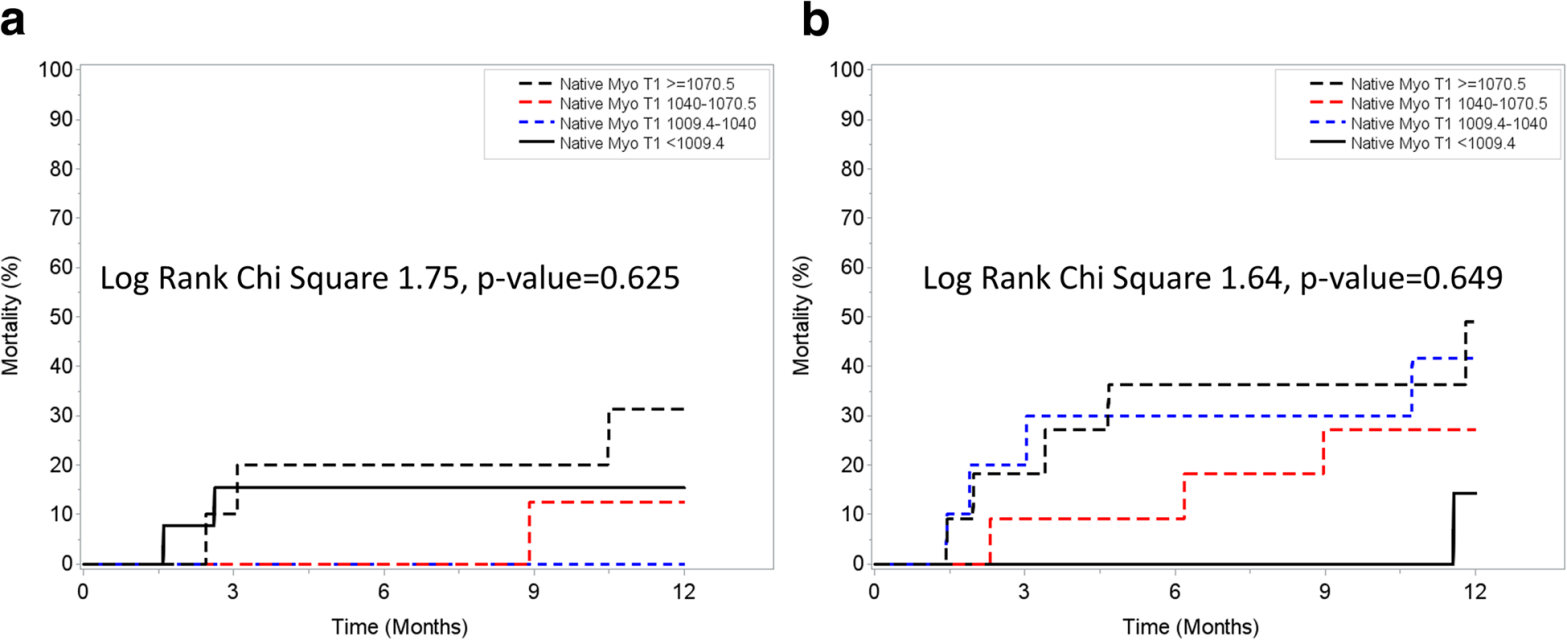
One year survival according to Native T1 quartiles stratified by aortic valve replacement (AVR) (Panel **a**) or Medical Therapy (Panel **b**)

**Fig. 7:**
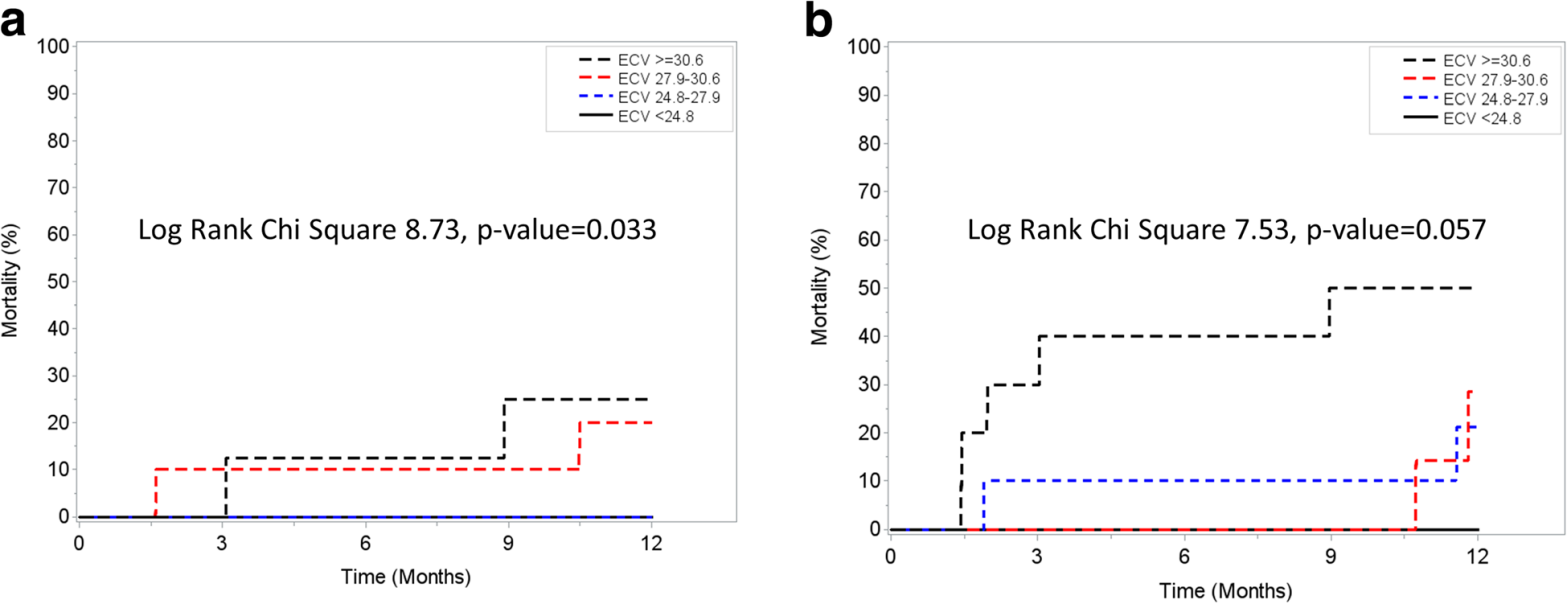
One year survival according to ECV quartiles stratified by AVR (Panel **a**) or Medical Therapy (Panel **b**)

Exploratory analysis of patients with native T1 and ECV values available for analysis demonstrated that although native T1 was not associated with the primary outcome (Fig. 4), ECV was associated with increased risk for all-cause mortality in a progressive dose-response manner (Fig. 5). Similar findings were observed when analysis was stratified based on the performance of AV replacement (Fig. 6, Panel A and Fig. 7, Panel A) vs. medical therapy (Fig. 6, Panel B and Fig. 7, Panel B). Of interest, for those patients with moderately-severe AS and managed conservatively without AVR, being in the lowest ECV quartile (ie: < 24.8%) was associated with a “protective” effect. Specifically, there were no deaths noted up to 12 months of follow-up for those patients in the lowest ECV quartile (Fig. 5 and Fig. 7, Panel A).

## Discussion

Our study has three main findings. First, the prevalence of CA in a large number of patients with moderate to severe AS is high, particularly among older (≥ 80 years) male patients where the prevalence is as high as 25%. These patients often presented with atrial fibrillation and low-flow, low-gradient AS physiology. Second, the combination of AS with CA is prognostically important as competing comorbidity; even among a subset of older AS patients, CA was associated with significantly increased 1-year all-cause mortality regardless of whether AVR occurred. Third, the presence of CA in elderly AS patients associates with all-cause mortality after adjustment of other potential comorbidities and confounders including AVR. Therefore, the coexistence of CA in moderate-severe AS may have important clinical implications for management and prognosis especially in the TAVR era.

Our findings build on the works of others. We highlight the relationship between CA prevalence and age. We show a higher prevalence (16%) of suspected CA in an older cohort (> 74 years) than Treibel and colleagues who reported a value of 5.6% in surgical AVR patients over 65 years of age [7]. We also included a larger mumber of patients with moderate and severe AS who did not necessarily undergo AVR, and we show adverse prognostic associations even with risk adjustment including STS-PROM and AVR among other variables. CA had once been considered a rare infiltrative disease. However, a proliferation of literature shows that CA is an underdiagnosed and under-recognized pathology in older adults, with prevalence approximately 25% in the general population, and higher in advanced age [1, 23].

Although myocardial biopsy remains the gold standard test for the diagnosis, over the last 5 years, advances in non-invasive cardiac imaging, including myocardial strain imaging [24], nuclear scintigraphy with the use of both 99^m^–Tc-DPD in Europe and 99^m^–Tc-PYP scans [25–27] in the United States, along with CMR with LGE imaging [11, 12, 14] and even cardiac computed tomography [28] have increased the capability of detecting this entity. ECV mapping as a measure of the interstitial expansion [29, 30] that is reproducible in other diseases [31] has the potential advantage of assessing serial changes of amyloid protein burden over time. ECV may be important for disease detection, following disease progression or monitoring response to therapy but this needs to be tested in a proper prospective randomized controlled trial. Multiple phase-2 studies currently under the way [32], highlight the rapid changes this field is undergoing opening the possibility for novel treatment strategies.

While prevalence of CA increase with age, screening for CA in AS patients remains uncertain. In men and women with moderate to severe AS, the optimal cut point for age above which CA is highly prevalent remains undefined. While we observed CA primarily in men over 80 years, other investigators have reported data that suggest a significant prevalence of wtATTR or CA in younger AS patients and female AS patients. For example, Nietlispach and colleagues found slightly more CA in women than men (3 of 5 cases) in an autopsy study post TAVR [2]. Treibel and colleagues also reported CA in AS patients as young as 69 years of age [7]. Their work also reveals the disease heterogeneity and spectrum of clinical presentation. For example, there were 4 patients with CMR findings consistent with AS, but who had myocardial biopsy demonstrating TTR amyloidosis. In one of those patients, despite severe AS and Perugini grade 1 on his DPD scintigraphy, had preserved global longitudinal strain, no increased LV wall thickness, normal ECV and normal NT-proBNP. In the selected subgroup of CA patients with ECV measurements, our values were comparable to theirs (mean ECV = 42 ± 15% vs. 39 ± 14%, *P\* = 0.71). As such, in AS patients, the optimal diagnostic algorithm for CA screening still needs to be sorted out, and further investigation is required.

Of note, patients with moderately-severe AS but ECV < 24.8% had no events at 12 months, irrespective of the treatment received (AVR vs. medical therapy). This “protective” ECV threshold is similar to the one recently reported by Schelbert et al. In a large, unselected cohort of patients undergoing clinical CMR at our institution, ECV < 25% was associated with no cardiovascular events such as heart failure hospitalizations or cardiovascular death up to 3 years of follow-up, irrespective of the LVEF [21]. Taken together, these findings should be considered hypothesis-generating. This might be particularly relevant in asymptomatic patients with moderate-severe AS but without ECV elevation where a more conservative approach might be considered. However, given the small subgroup available for this exploratory analysis, further validation of these observations is required in larger cohorts of AS patients.

With the increased longevity of the population and the growing number of elderly patients, the convergence of these two aging processes – namely calcific AS and CA might represent an important intersection where a careful comprehensive evaluation and treatment planning is needed.

### Limitations

Our study has limitations. First, relying predominantly on LGE imaging pattern to detect CA, we may have misclassified patients’ amyloidosis status. A novel algorithm recently proposed by Gillmore and colleagues using upfront nuclear scintigraphy scan for patients with heart failure and clinically suspected findings on CMR [26] is currently what has been used in our center starting in late 2015 when Tc-PYP scan became available. We acknowledge that the absence of endomyocardial biopsy data in these patients prevented definitive diagnosis of CA and subtyping. However, endomyocardial biopsy is not only invasive for these patients who have a high burden of comorbidities and frailty but can also be associated with sampling error. We believe that the totality of the clinical evidence reasonably supported its diagnosis which was accepted by clinicians caring for the patient. Whether the combined use of LGE pattern and ECV measurements could improve the sensitivity to detect CA is unknown needs to be tested prospectively. Nonetheless, regardless of the potential misclassification we obtained significant findings and results.

Second, this single-center study of the referred CMR patients may involve selection bias that could influence disease prevalence estimates. Our CMR eligibility criteria exclude patients with cardiac devices (defibrillators, pacemakers) or renal dysfunction (i.e., estimated glomerular filtration rate < 30 ml/min/1.73 m^2^ given the need for gadolinium contrast. Yet, these exclusions may lower prevalence estimates, since both chronic kidney insufficiency and of arrhythmia/conduction disease requiring implantable cardiac devices increase with aging, the potential initial overestimation of a “selected CMR cohort” might be counter-balanced by the relative contra-indications for CMR study eligibility and performance. Supporting that observation is the recent publication by Castaño who systematically obtained Tc-PYP scanning in patients who received TAVR (mean age = 84 ± 6 years) [33]. The authors identified the prevalence of 16% for ATTR-CA, which is similar prevalence noted to our cohort when we considered those above median cohort age of 74 years. Nonetheless, our cohort represents a selected group of AS patients who received CMR for certain clinical indications.

Third, data on other clinical outcomes such as cardiac-specific mortality, heart failure hospitalizations, were not always available. Our limited sample size did not permit subgroup analyses yet it was sufficiently large to permit multivariable survival analysis for the first time. Lastly, CMR techniques using gadolinium are not applicable to some of these patients with advanced renal dysfunction. Evolving CMR quantitative capabilities using native (noncontrast) T1 mapping [34, 35] and ECV may prove useful but require further validation in larger cohorts.

It is important to note that not all providers embraced CMR to characterize myocardial fibrosis in the myocardium of AS patients (as articulated by Dweck et al. [36]), which underscores the potential for referral bias.

## Conclusions

Suspected CA by CMR appears prevalent among older individuals in particular for males with low-flow, low-gradient AS. In our cohort, 25% of octogenarians presenting with cardiac symptoms and AS were found to have concomitant CA. CMR diagnosis of presumed CA associates with all-cause mortality. The coexistence of these 2 conditions in the same patient, has several important clinical implications in the diagnosis, management and prognosis. Screening for CA in older AS patients and optimal treatment strategies in those with CA warrant further investigation, especially in the era of TAVR. Larger, multicenter prospective studies are urgently required for these patients to better inform medical decision making and patient stratification for future therapeutic interventions.

## Abbreviations

AS: aortic stenosis
AV: aortic valve
AVA: aortic valve area
AVR: aortic valve replacement
CA: cardiac amyloidosis
CI: confidence interval
CMR: cardiovascular magnetic resonance
ECV: extracellular volume fraction
IQR: interquartile range.
LGE: late gadolinium enhancement
LV: left ventricle/left ventricular
LVEF: left ventricular ejection fraction
LVOT: left ventricular outflow tract
MOLLI: modified Look-Locker inversion recovery
PSIR: phase sensitive inversion recovery
STS-PROM: Surgical Thoracic Society Predicted Risk of Mortality Score
TAVR: transcatheter aortic valve replacement
TTE: Transthoracic echocardiography
VTI: velocity time interval
wtATTR: wild-type transthyretin-related cardiac amyloidosis
